# Spectrum of Non-Motor Symptoms in Parkinson's Disease

**DOI:** 10.7759/cureus.13275

**Published:** 2021-02-11

**Authors:** Maithrayie Kumaresan, Safeera Khan

**Affiliations:** 1 Neurology, California Institute of Behavioral Neurosciences & Psychology, Fairfield, USA; 2 Internal Medicine, California Institute of Behavioral Neurosciences & Psychology, Fairfield, USA

**Keywords:** parkinson' s disease, non-motor symptoms, sleep disorders, olfactory dysfunction, gastrointestinal disorders

## Abstract

Parkinson's disease is predominantly classified as a movement disorder. Beyond the textbook definition of rigidity, tremors, and bradykinesia, Parkinson's disease encompasses an entire entity of non-motor symptom complexes that can precede the motor features by many years. Despite their significant clinical importance, the awareness of non-motor symptoms is quite negligible. Sleep disorders, gastrointestinal dysfunction, olfactory disturbances, anxiety, and depressive episodes are some of the most common non-motor presentations. The wide-spread occurrence of olfactory symptoms and the low cost of the assessment, is favoring olfactory dysfunction as a potential biomarker in Parkinson's. Sleep disorders may manifest before the motor and autonomic symptoms and might be linked to concomitant sleeping disorders like insomnia, REM sleep disorders, restless leg syndrome, narcolepsy, or obstructive sleep apnea. Non-motor symptoms can deteriorate the quality of life in Parkinson’s patients. Early detection of non-motor symptoms can help in the diagnosis of Parkinson’s disease and can fairly improve the survival and prognosis of these patients.

## Introduction and background

The characteristic feature of Parkinson’s disease (PD) is the loss of dopaminergic neurons in the substantia nigra and the presence of alpha-synuclein protein Lewy body, named after the German neuropathologist Friedrich Heinrich Lewy. Parkinson’s disease affects two per 1000 population at any given time. The prevalence of PD increases with age and is more common in men than in women [1]. The triad of parkinsonism is often defined by rigidity, bradykinesia, and tremors. However, non-motor symptoms (NMS) in PD is a common occurrence and was recognized first in 1817 by James Parkinson in his essay "Shaking Palsy" [2].

NMS can manifest at any stage of the disease, sometimes even in the early and pre-motor phases. Around 30 different NMS have been highlighted in PD [2]. Braak and colleagues proposed that the varying degrees of synuclein pathology depicted a temporal sequence of events that could be classified into six stages [3]. They postulated that environmental neurotoxins gain entry into the gastrointestinal tract via the nasal cavity, initiating Lewy body pathogenesis. Similarly, there was another hypothesis stating the spread of Lewy pathology (LP) from the peripheral to the cerebrospinal nervous system.

Motor impairments in PD may be heralded by non-motor symptoms such as depressive disorders, olfactory deficit, rapid eye movement (REM) sleep disorders, constipation, and anxiety, by even up to ten years. Sleep disturbances are highly prevalent in patients with PD and are indicators of poor caliber of life. The assessment of sleep disorders in these patients is complex. As a host of primary medical disorders, psychiatric conditions, increasing age, or the neuropathophysiology of PD itself may affect the sleep cycle [4]. Olfactory deficits are common and often supersede the diagnosis of PD by years, signifying prior initiation of Lewy body pathology. Olfactory dysfunction coupled with other non-motor features of PD and may serve as an indicator of cognitive decline [5]. Despite their vast clinical relevance, NMS is often overlooked by physicians and dismissed by patients. Usage of a patient-based screening tool such as a non-motor symptom questionnaire (NMS quest) draws attention to and strengthens the early management of NMS [6]. For the effective care of PD patients, a multidisciplinary approach encompassing both pharmacological and non-pharmacological treatment is mandatory. In this literature study of 50 review articles, the pathophysiology and features of three main non-motor symptoms have been highlighted. 

## Review

The three most common non-motor symptoms in Parkinson's disease have been analyzed below from 50 review studies. All studies highlight the importance of non-motor symptoms in the early detection of Parkinson's disease and talk about the precedence of NMS over motor symptoms. On-going clinical trials and existing hypotheses on the pathophysiology of NMS have also been elicited.

Gut dysfunction in Parkinson’s disease

Gastrointestinal (GI) or gut dysfunction in PD can be caused by both motor and non-motor (dysautonomic) impairment. It has now been established that gut disturbances in patients with PD are quite a frequent occurrence [7]. Around 60% to 80% of the PD patients present with GI changes which serve as an early indicator for assessing the quality of life [8]. Gut dysfunctions such as sialorrhoea, hypogeusia, dysphagia, delayed gastric emptying, weight loss, and dyschezia present early in the disease is relatively high frequencies [9]. The gut may serve as a pathway for the PD toxin to reach the central nervous system (CNS). There is increasing evidence suggesting the gut microbiome’s diverse and potent influence over CNS-related conditions apart from PD, including autism spectrum disorders and Alzheimer's disease [10]. Enteroendocrine cells are specialized intestinal cells that express various peptides/hormones and act as signaling channels, via which the gut microbiota communicates with the cerebrospinal nervous system through the X^th^ cranial nerve (vagus) [11].

**Figure 1:**
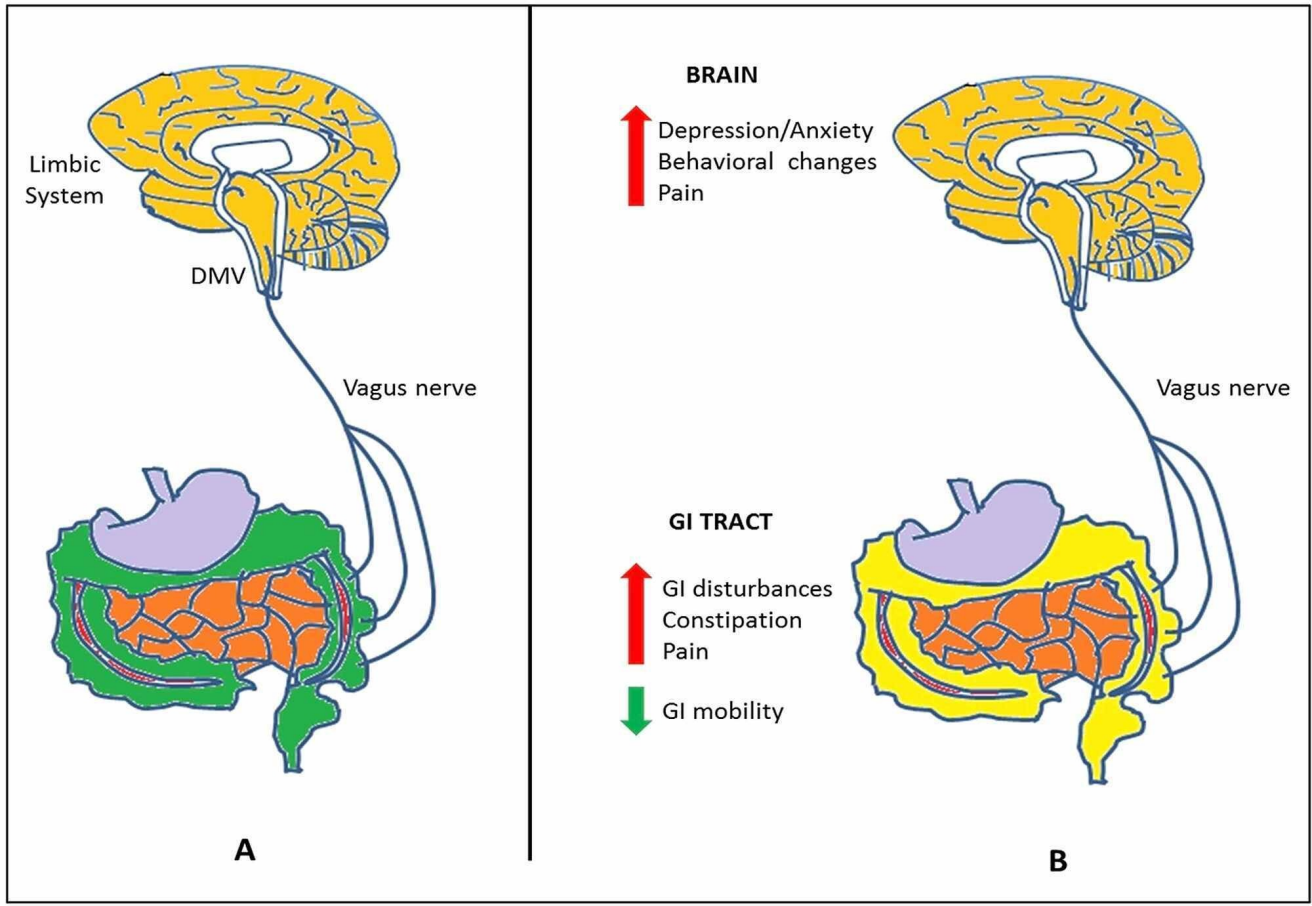
The brain-gut axis in Parkinson’s disease. (A) Two-way communication between the brain and the gut via the vagus nerve. (B) Non-motor symptoms of Parkinson’s disease.

Animal trials have illustrated that α-synuclein bundles can disseminate in a prion-like style via microtubule-associated transport along the axons and spread to neighboring brain regions. In short, alpha-synuclein functions as a prion-like protein, which can act as seeds for further aggregation and undergo transport along axons transferring to subsequent neurons and eventually cause neuronal degeneration in the substantia nigra. Though studies have linked vagotomy with a decreased risk of Parkinson's in humans, conclusive evidence to support the prion hypothesis and the cerebral origin of alpha-synuclein aggregates is still amiss. It is also to be noted that patients with neural grafting in Parkinson’s disease have developed alpha-synuclein pathology. Alpha-synuclein from PD patients is also known to cause nigrostriatal degeneration in mice and other mammals (Figure 1, Table 1) [12].

**Table 1:** Previous studies about gut dysfunction in Parkinson’s disease. PD- Parkinson's disease; GI- gastro-intestinal; CCK- cholecystokinin; EEC- enteroendocrine cell; ENS- enteric nervous system

Author	Year of publication	Type of study	Purpose of the study	Intervention studied	Result/conclusion
Caputi and Grion [13]	2018	Review	Newer treatment	Microbiome-gut-brain axis and Toll-like receptor ligands in PD.	Prebiotics, probiotics, and synbiotics can be used effectively for the prophylaxis and treatment of neurodegeneration. Probiotics produce anti-inflammatory cytokines through Toll-like receptors and can suppress inflammatory activity.
Mukherjee et al. [14]	2016	Review	The study focused on various gut dysfunctions associated with PD	Microbiota and GI system	The study revealed that in PD, the gut is involved extensively in the pathophysiology of the disease.
Cryan et al. [15]	2019	Review	To study the role of microbiota in regulating the gut-brain axis.	Microbiota-gut-brain axis	The gut microbiota is important for adequate maintenance of cerebral activity.
Perez-Pardo et al. [16]	2017	Review	To study the gut-brain axis in Parkinson's disease as well as identifying the possibilities for food-based therapies	Alpha-synuclein aggregates and enteric nervous system involvement.	The study supports the theory that PD could originate in the gut
Liddle [17]	2018	Research article	Possibility of PD originating in the gut in light of the recent unveiling of an enteroendocrine cell – neural circuit and the discovery of α-synuclein in enteroendocrine cells.	Immuno-histological staining of a CCK type EEC cell.	Ingested toxins in the gut microbiome can induce α-synuclein aggregation and subsequently cause PD.
Pfeiffer [18]	2018	Review	To determine the potential of gut microbiota in the genesis of PD.	Gut microbiome	Small intestinal dysfunction may play a role in Parkinson's disease, especially concerning erratic responses to Parkinson medication
Sharma et al. [19]	2019	Review	The use of probiotics and prebiotics as a new treatment modality to retain normal gut flora and prevent PD-like symptoms.	Gut-brain axis	The alteration of gut flora leading to inflammation of the enteric system and aggravating prion manner synuclein pathology leading to PD.
Klingelhoefer and Reichmann [20]	2017	Review	Clinical and pathological review to substantiate the theory that PD originates in the gut and spreads via trans-synaptic cell-to-cell transfer through the autonomic nervous systems to the central nervous system.	GI and the enteric nervous system.	Rostro-cranial transmission of PD from the olfactory bulb/ ENS to the substantia nigra, questioning the involvement of environmental triggers in the pathogenesis of the disease.
IJzendoorn and Derkinderen [21]	2019	Review	The intestinal barrier in PD.	Microbiota and the enteric nervous system	Studying the possibility of intestinal permeability in neurodegenerative conditions.

Olfactory disorders in Parkinson's disease

Olfactory dysfunction is a distinct early feature of Parkinson's disease. Research has indicated that >95% of PD patients have impairment of olfactory function. Deficits in the sense of smell may precede clinically relevant motor symptoms by many years and can be used as an early assessment tool for Parkinson's disease in asymptomatic individuals [22]. Ample studies are looking into olfactory disorders in the pre-motor stages of PD. The Honolulu-Asia Aging Study, a longitudinal study following 2267 men between 71 years and 95 years of age without known PD, reported an odds ratio of 5.2 for PD development in subjects within the lowest quartile odor identification test after a four-year follow-up [23].

**Figure 2:**
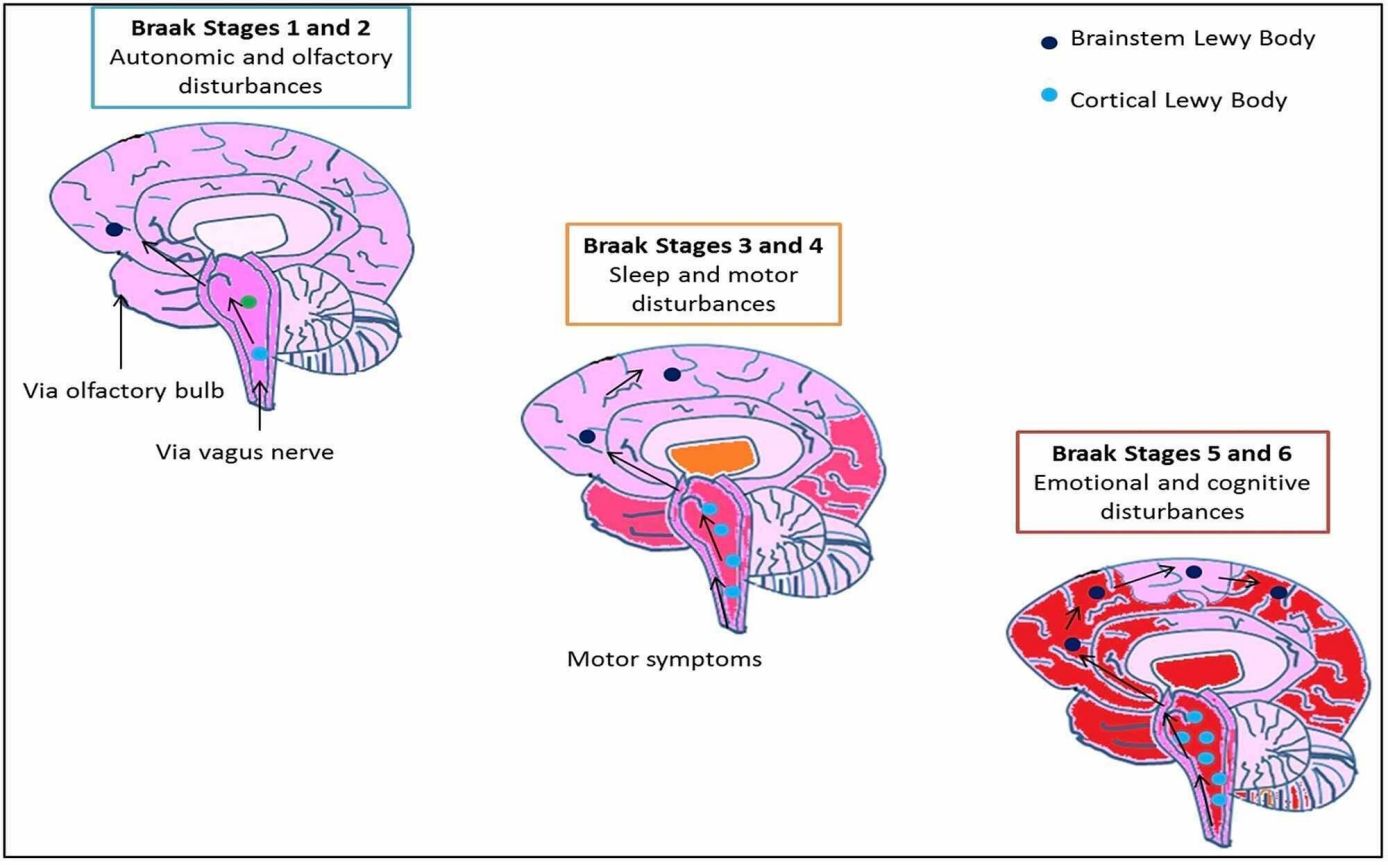
Braak staging steps 1-6. The pink shading represents the pattern of spread of alpha-synuclein from the olfactory region to the cortex.

The presence of Lewy bodies (alpha-synuclein as the main component) in the substantia nigra and Lewy neuritis is characteristic of Parkinson’s disease. Braak et al. believed that Lewy body pathogenesis originated in the olfactory bulb and dorsal motor nucleus of the tenth cranial nerve [24]. Alpha-synuclein deposition in olfactory structures occurs before deposition in the substantia nigra This happens in the initial stages of the Braak hypothesis. By Braak stage 3, LB pathology involves the medulla and pontine tegmentum by moving up the brainstem [25]. Motor symptoms become prominent at this stage. Neuropathological studies in normal human brains identified Lewy body isolates. Braak documented that when only a part of the brain is affected with incidental Lewy bodies, it was most likely to be the olfactory bulb [26]. Incidental Lewy bodies have been studied in the olfactory bulb, anterior olfactory nucleus, and the cortices of the olfactory tract in patients with neurodegeneration. The beginning of the Lewy pathology can be traced to the thin olfactory nerve sheath; it then spreads to the olfactory bulb's glomeruli from where it projects to the mitral and tufted cells. The pathology extends from the dendrites of the mitral cells to the nucleus of the amygdala in the cortex [27]. Early alpha-synuclein deposition and neuronal degeneration prior to the appearance of motor features, sometimes even as early as four years, can be traced back to this part of the cortex. Upcoming functional genomics studies are identifying alteration in the cortical olfactory and taste receptors in PD patients (Figure 2, Table 2).

**Table 2:** Previous studies about olfactory disorder in Parkinson’s disease. PD- Parkinson's disease; PET- positron emission tomography; EEG- electroencephalography; MMSE: mini-mental state examination

Author	Year of publication	Type of study	Purpose of the study	Intervention studied	Result/conclusion
Roos et al. [28]	2019	Research article	To evaluate the relationship between hyposmia and motor features of PD.	MMSE	Hyposmia in PD is directly linked to the degree of nigrostriatal dopaminergic degradation.
Marin et al. [29]	2018	Review	Olfactory function assessment and quantification.	Olfaction as a clinical marker for neurodegenerative diseases.	Understanding the mechanisms underlying olfactory dysfunction and its association with neurodegenerative disorders.
Oh et al. [30]	2018	Research article	Specialized MRI guided techniques to assess the status of the dopamine active transporter in the cortex.	MRI, PET image	Importance of gut microbiota to maintain normal brain functions.
Leonhardt et al. [31]	2019	Research article	Assessing the awareness of olfactory impairments in Parkinson’s.	Odor identification test and the subjective olfactory capability.	Anosmia and decreased respiratory function could serve as a marker for cognitive decline in PD.
Fullard et al. [32]	2017	Research article	Recognizing early biomarkers in Parkinson’s.	Olfaction as a potential biomarker for early recognition of Parkinson’s.	Linking early biomarkers to cognitive decline function.
Tarakad and Jankovic [33]	2017	Review	To determine the association of anosmia and ageusia in Parkinson's Disease	Evaluation of olfactory thresholds, smell identification and discrimination, and olfactory memory.	Olfactory disorders are more prominent in Parkinson’s disease compared to other neurodegenerative conditions.
Taniguchi and Takeda [34]	2017	Review	To study the olfactory dysfunction as a clinically detectable motor sign for PD	Smell tests.	Smell tests seem to be a feasible diagnostic tool for early PD and a potentially useful biomarker for Parkinson's disease dementia.
Domellöf et al. [35]	2017	Research article	The presentation and evolution of olfactory deficits from the time of PD diagnosis.	Brief smell identification test.	The risk of dementia increased ten times manifold in PD patients with anosmia.
Iannilli et al. [36]	2017	Research article	Studying the co-relation of EEG with olfactory function assessment.	64-channel electroencephalography and psychophysical tests.	The difference in the mechanism of olfactory dysfunction in patients with and without Parkinson’s.

Sleep disorders in Parkinson's disease

Sleep disorders are the most common NMS associated with PD and have been studied in 38-98% of PD patients [37]. James Parkinson himself mentioned the poor sleep quality in PD patients in his famous monograph about the disease. Sleep disorders like olfactory dysfunctions can occur years before the appearance of motor features [38]. Decreased sleep quality, daytime fatigue, and nocturnal awakenings characterize sleep in PD [39]. These disorders may precede motor and autonomic symptoms like akinesia, rigidity, dystonia, and nocturia and might be linked to concomitant sleeping disorders like insomnia, REM sleep disorders, narcolepsy, or sleep apnea. Daytime somnolence, sudden sleep attacks, and other diurnal sleep disturbances can be caused by the levodopa treatment or the neuropathology of PD itself. The early recognition and management of sleep disorders in PD are essential because of their negative impact on life quality. Studies have reported an early morning “sleep benefit” (improved motor function on waking up) prior to medication intake in PD patients [40]. Högl et al. described that the graph linking levodopa concentrations and polysomnographic studies shared a similar pattern between PD patients with and without the sleep benefit. However, PD patients with sleep benefit demonstrated a different response curve to levodopa - the immensity of motor function deterioration after levodopa intake was higher in PD patients with the sleep benefit compared to patients without it [41]. Previous studies have also reported a high prevalence of obstructive sleep apnea (OSA) in PD patients (approximately 20-60%). The hypothesis surrounding OSA is PD related loss of functioning neurons in myofibers and their subsequent atrophy, degeneration of the peripheral nerves innervating the oropharyngeal muscles, alpha-synuclein deposition in the fibers of the vagus nerve, supplying the laryngeal and pharyngeal muscles, and the episodic upper airway movement disorders caused by nocturnal dyskinesia may play a pivotal role in the development of obstructive sleep apnea. Nevertheless, the clinical relevance of OSA in PD remains a topic for further debate (Table 3).

**Table 3:** Previous studies about sleep disorders in Parkinson’s disease. PD- Parkinson's disease; REM- rapid eye movement; SDB- sleep-disordered breathing; RBD- REM sleep behavior disorder; EDS- excessive daytime sleepiness

Author	Year of publication	Type of study	Purpose of the study	Intervention studied	Result/conclusion
Chahine et al. [42]	2017	Review	Sleep wakefulness cycle in PD.	A systematic review on REM sleep behavior disorders, periodic limb movements, sleep-disordered breathing, and circadian rhythm disorders.	There is an influence of the medications used in the treatment of PD on the sleep cycle.
Doręgowska and Rudzińska-Bar [43]	2019	Review	The study discussed sleep physiology, different sleep dysfunctions such as REM sleep behavior disorder, EDS, insomnia, obstructive sleep apnea syndrome, and their clinical manifestations.	Sleep physiology	There is a coexistence of various non-motor symptoms such as pain, depression, or nocturia with sleeping disorders
Falup-Pecurariu and Diaconu [44]	2017	Review	Detection and management of sleep disorders in PD.	Sleep recording techniques-actigraphy or polysomnography.	Insomnia, daytime somnolence, REM sleep disorders, and restless leg syndrome are all encountered in PD.
Shen and Liu [45]	2018	Editorial	The study discussed the present status and future prospects of sleep disorders in PD.	Genetic studies of sleep disorders.	It’s debatable if sleep disorders enhance PD progression.
Crosta et al. [46]	2017	Research article	The study looked into the occurrence of sleep-disordered breathing in a large consecutive outpatient series	Epworth sleepiness scale, polysomnography	The study suggested an increased frequency of obstructive sleep apnea syndrome and other SDB in PD and parkinsonism.
Bollu and Sahota [47]	2017	Review	To study the various sleep disorders seen in PD patients	Sleep fragmentation, wakefulness after sleep onset	The study helps understand sleep issues in PD, which can help identify them early and result in optimal management.
Barone and Henchcliffe [48]	2018	Review	The study looked into rapid eye movement sleep behavior disorder and its association with alpha-synucleinopathies.	Alpha-synucleinopathy	RBD alone is now recognized to be a significant causative factor of neuropathological ailments especially Lewy body dementia.
Sobreira-Neto et al. [49]	2017	Research article	Frequency study of major sleep disorders in Parkinson's disease	Standardized scales- Epworth Sleepiness Scale, PD questionnaire, Pittsburgh sleep quality index.	The impact of day time somnolence on the quality of life.
Kim et al. [50]	2018	Review	To probe the significance of REM sleep behavior disorder as a prognostic factor in PD	Baseline RBD.	Studying the co-relation of RBD with the prognosis and mortality of PD patients.

## Conclusions

Despite the growing awareness of PD's non-motor symptoms, most clinical trials still focus on motor symptoms as the primary consequence. The prodromal NMS phase's basis relies on the Lewy pathology hypothesis, which starts outside the substantia nigra. Though backed by in vitro, in vivo, and clinical evidence, this hypothesis isn’t widely accepted. Gut dysfunction, depression, REM sleep disorders amongst the many NMS can prove to be seriously disabling and can deteriorate the quality of life in Parkinson’s patients. Early recognition and quantitation of NMS can thus aide in the management and sometimes even the early diagnosis of Parkinson’s disease.

## References

[1] Tysnes OB, Storstein A (2017). Epidemiology of Parkinson’s disease. J Neural Transm.

[2] Mehndiratta M, Garg RK, Pandey S (2011). Nonmotor symptom complex of Parkinson’s disease-an under-recognized entity. J Assoc Physicians India.

[3] Burke RE, Dauer WT, Vonsattel JPG (2008). A critical evaluation of the Braak staging scheme for Parkinson's disease. Ann Neurol.

[4] Menza M, Dobkin RD, Marin H, Bienfait K (2010). Sleep disturbances in Parkinson's disease. Mov Disord.

[5] Fullard ME, Morley JF, Duda JE (2017). Olfactory dysfunction as an early biomarker in Parkinson’s disease. Neurosci Bull.

[6] Wishart S, Macphee GJ (2011). Evaluation and management of the non-motor features of Parkinson’s disease. Ther Adv Chronic Dis.

[7] Pfeiffer RF (2018). Gastrointestinal dysfunction in Parkinson’s disease. Curr Treat Options Neurol.

[8] Poirier AA, Aubé B, Côté M, Morin N, Paolo TD, Soulet D (2016). Gastrointestinal dysfunctions in Parkinson’s disease: symptoms and treatments. Parkinsons Dis.

[9] Sung H Y, Park J W, Kim J S (2014). The frequency and severity of gastrointestinal symptoms in patients with early Parkinson’s disease. J Mov Disord.

[10] Santos SF, De Oliveira HL, Yamada ES, Nerves BC, Pereira A Jr (2019). The gut and Parkinson’s disease-a bidirectional pathway. Front Neurol.

[11] Christensen J (1985). The response of the colon to eating. Am J Clin Nutr.

[12] Latorre R, Sternini C, De Giorgio R (2016). Enteroendocrine cells: a review of their role in brain-gut communication. Neurogastroenterol Motil.

[13] Caputi V, Giron MC (2018). Microbiome-gut-brain axis and toll-like receptors in Parkinson’s disease. Int J Mol Sci.

[14] Mukherjee A, Biswas A, Das SK (2016). Gut dysfunction in Parkinson's disease. World J Gastroenterol.

[15] Cryan JF, Riordan KJ, Cowan CS (2019). The microbiota-gut-brain axis. Physiol Rev.

[16] Perez-Pardo P, Kliest T, Dodiya HB, Broersen LM, Garssen J, Keshavarzian A, Kraneveld AD (2017). The gut-brain axis in Parkinson's disease: possibilities for food-based therapies. Eur J Pharmacol.

[17] Liddle RA (2018). Parkinson's disease from the gut. Brain Res.

[18] Pfeiffer RF (2018). Gastrointestinal dysfunction in Parkinson’s disease. Curr Treat Options Neurol.

[19] Sharma S, Awasthi A, Singh S (2019). Altered gut microbiota and intestinal permeability in Parkinson’s disease: pathological highlight to management. Neurosci Lett.

[20] Klingelhoefer L, Reichmann H (2017). The gut and nonmotor symptoms in Parkinson's disease. Int Rev Neurobiol.

[21] Van IJzendoorn SC, Derkinderen P (2019). The intestinal barrier in Parkinson’s disease: current state of knowledge. J Parkinsons Dis.

[22] Haehner A, Hummel T, Reichmann H (2011). Olfactory loss in Parkinson's disease. Parkinsons Dis.

[23] Abbott RD, Ross GW, White LR (2003). Environmental, life-style, and physical precursors of clinical Parkinson’s disease: recent findings from the Honolulu-Asia Aging Study. J Neurol.

[24] Braak H, Del Tredici K, Rüb U, De Vos R, Steur E, Braak E (2003). Staging of brain pathology related to sporadic Parkinson’s disease. Neurobiol Aging.

[25] Hawkes CH, Tredici K, Braak H (2010). A timeline for Parkinson's disease. Parkinsonism Relat Disord.

[26] Beach TG, Adler CH, Lue L (2009). Unified staging system for Lewy body disorders: correlation with nigrostriatal degeneration, cognitive impairment and motor dysfunction. Acta Neuropathol.

[27] Kohl Z, Schlachetzki C, Feldewerth J (2017). Distinct pattern of microgliosis in the olfactory bulb of neurodegenerative proteinopathies. Neural Plast.

[28] Roos DS, Twisk JW, Raijmakers PG, Doty R, Berendse HW (2019). Hyposmia as a marker of (non-) motor disease severity in Parkinson’s disease. J Neural Transm.

[29] Marin C, Vilas D, Langdon C (2018). Olfactory dysfunction in neurodegenerative diseases. Curr Allergy Asthma Rep.

[30] Oh YS, Kim JS, Hwang EJ, Lyoo CH (2018). Striatal dopamine uptake and olfactory dysfunction in patients with early Parkinson's disease. Parkinsonism Relat Disord.

[31] Leonhardt B, Tahmasebi R, Jagsch R, Pirker W, Lehrner J (2019). Awareness of olfactory dysfunction in Parkinson’s disease. Neuropsychology.

[32] Fullard ME, Morley JF, Duda JE (2017). Olfactory dysfunction as an early biomarker in Parkinson’s disease. Neurosci Bull.

[33] Tarakad A, Jankovic J (2017). Anosmia and ageusia in parkinson's disease. Int Rev Neurobiol.

[34] Taniguchi S, Takeda A (2017). Olfactory dysfunction. Nihon Rinsho.

[35] Domellöf ME, Lundin K-F, Edström M, Forsgren L (2017). Olfactory dysfunction and dementia in newly diagnosed patients with Parkinson's disease. Parkinsonism Relat Disord.

[36] Iannilli E, Stephan L, Hummel T, Reichmann H, Haener H (2017). Olfactory impairment in Parkinson’s disease is a consequence of central nervous system decline. Eur Neurol.

[37] Tandberg E, Larsen JP, Karlsen K (1999). Excessive daytime sleepiness and sleep benefit in Parkinson's disease: a community‐based study. Mov Disord.

[38] Melka D, Tafesse A, Bower JH (2019). Prevalence of sleep disorders in Parkinson’s disease patients in two neurology referral hospitals in Ethiopia. BMC Neurol.

[39] Peeraully T, Yong MH, Chokroverty S, Tan E (2012). Sleep and Parkinson's disease: a review of case‐control polysomnography studies. Mov Disord.

[40] Suzuki K, Miyamoto M, Miyamoto T, Iwanami M, Hirata K (2011). Sleep disturbances associated with Parkinson's disease. Parkinsons Dis.

[41] Höl B, Arevalo G, Garcia S, Scipioni O, Rubio M, Blanco M, Gershanik OS (1998). A clinical, pharmacologic, and polysomnographic study of sleep benefit in Parkinson's disease. Neurology.

[42] Chahine LM, Amara AW, Videnovic A (2017). A systematic review of the literature on disorders of sleep and wakefulness in Parkinson's disease from 2005 to 2015. Sleep Med Rev.

[43] Doręgowska M, Rudzińska-Bar M (2019). Sleep disorders in Parkinson's disease. Wiad Lek.

[44] Pecurariu C, Diaconu S (2017). Sleep dysfunction in Parkinson's disease. Int Rev Neurobiol.

[45] Shen Y, Liu C-F (2018). Sleep disorders in Parkinson's disease: present status and future prospects. Chin Med J.

[46] Crosta F, Desideri G, Marini C (2017). Obstructive sleep apnea syndrome in Parkinson’s disease and other parkinsonisms. Funct Neurol.

[47] Bollu PC, Sahota P (2017). Sleep and Parkinson's disease. Mo Med.

[48] Barone DA, Henchcliffe C (2018). Rapid eye movement sleep behavior disorder and the link to alpha-synucleinopathies. Clin Neurophysiol.

[49] Sobreira-Neto MA, Pena-Pereira MA, Sobreira EST, Chagas MHN, Fernandes RMF, Tumas V, Eckeli AL (2017). High frequency of sleep disorders in Parkinson’s disease and its relationship with quality of life. Eur Neurol.

[50] Kim Y, Kim YE, Park EO, Kim HJ, Jeon B, Shin CW (2018). REM sleep behavior disorder portends poor prognosis in Parkinson’s disease: a systematic review. J Clin Neurosci.

